# Noise Suppression for Direction of Arrival Estimation in Co-located MIMO Sonar

**DOI:** 10.3390/s19061325

**Published:** 2019-03-16

**Authors:** Xue Cheng, Yingmin Wang

**Affiliations:** School of Marine Science and Technology, Northwestern Polytechnical University, Xi’an 710072, China; chengxue0505@mail.nwpu.edu.cn

**Keywords:** direction-of-arrival estimation, MIMO sonar, noise suppression, covariance matrix, Toeplitz

## Abstract

Noise suppression capacity in multiple-input multiple-output (MIMO) sonar signal processing is derived under the assumption of white Gaussian noise. However, underwater noise mainly includes white Gaussian noise and colored noise. There exists a certain correlation between the noise signals received by each MIMO sonar array element. The performance of traditional direction-of-arrival (DOA) estimation methods decreases obviously in complex marine noise. In this paper, we propose a marine environment noise suppression method for MIMO applied to multiple targets’ DOA estimation. The noise field can be decomposed into a symmetric noise component and an asymmetric noise component. We use the covariance matrix imaginary component to pre-estimate the signal sources, then use the dimension reduction transformation to reconstruct the real component of the covariance matrix. The Toeplitz technique is utilized to reduce the correlation of the reconstructed covariance matrix. Thus, the subspace decomposition-based techniques such as multiple signal classification (MUSIC) can be used for multiple targets’ DOA estimation. To reduce the computational complexity of the methods, search-free direction-finding techniques such as the estimation of signal parameters via rotational invariance techniques (ESPRIT) can be utilized. As a result, the proposed methods can achieve better direction-finding performance in the condition of limited snapshots with lower computational cost. The corresponding Cramer-Rao bound (CRB) is deduced and the signal-to-noise ratio (SNR) gain obtained by dimension reduction processing is discussed. Simulation results also show the superiority of the proposed method over the existing methods.

## 1. Introduction

Multiple-input multiple-output (MIMO) sonar is a system that consists of the transmit array and the receive array. Both arrays employ multiple sensors. The transmit array emits orthogonal waveforms and the receive array completes the echo signal acquisition [1,2,3]. The MIMO array can be mainly classified into two types. One type is equipped with separated sensors [4,5,6,7,8]. This kind of MIMO sonar system utilizes widely separated sensors to gain spatial diversity. The large aperture arrays at both the transmitter and receiver enable the MIMO sonar to view different aspects of a target [9]. The other type is equipped with co-located sensors [10,11,12]. This kind of MIMO sonar employs arrays of closely spaced sensors to view the same side of one target from the same angle. In this scenario, it is assumed that the source is the point target in the far field of MIMO sonar. Additionally, the transmitted signals are normally assumed to be narrowband. Hassanien et al. [13] assumed the point target signal model. Under this circumstance, since the receiver gathers the multiple independent waveforms, the diversity of the waveform is used to increase the receiver virtual aperture.

Direction-of-arrival (DOA) estimation of multiple targets is corrupted by complex marine noise at the receiver sensors. DOA estimation accuracy improvement is one of the most important issues to be overcome in the application of MIMO sonar [14,15]. Many DOA estimation techniques have been proposed for the MIMO array [16,17,18]. The concept of phased-MIMO array is introduced in References [13,19,20,21]. It is shown that a tradeoff between phased-array and MIMO array may help to gain high-angle resolution. By using proper transmit beam space design, it is possible to satisfy the desired property. In comparison to the previous methods, Luo et al. [9,22] used the iterative approach to improve the DOA estimation super-resolution. A joint transmitter and receiver optimization approach was proposed. It is very important to enhance the direction-finding ability at a low signal-to-noise ratio (SNR) in the situation of a complex marine environment. The methods proposed above are applicable to the background noise as white Gaussian noise. In the case of complex marine environment noise, in order to avoid the influence of colored noise on MIMO direction finding, several techniques are proposed to suppress the spatial colored noise that exists in the marine noise environment [23,24,25,26,27]. The approach adopted by Zhou et al. [23] is based on the characteristic that higher order cumulants are not sensitive to Gaussian noise. This helps to avoid the effect of the colored noise component on the accuracy of direction finding. By utilizing an angle estimation method that uses both the Estimating Signal Parameters via rotational invariance techniques (ESPRIT) and singular value decomposition (SVD) of the cross-correlation matrix, spatial colored noise suppression can be effective for three or more transmitters [24]. A novel algorithm is proposed by combining the canonical correlation decomposition (CCD) and the shift-invariance properties of the various steering matrices [25]. Recently, the tensor subspace-based versions of the cross-correlation methods have been derived [26,27]. Nevertheless, all the above methods are only applicable to the distributed MIMO array. If we use the co-located MIMO array, these methods will be unable to meet the accuracy requirements of the DOA estimation. On the other hand, the DOA estimation method proposed by Xia et al. [28] is based on the real part reconstruction of the covariance matrix. It solves the target direction fuzzy problem coming from the bilateral spectrum. The lake experiment is carried out to verify its effectiveness. The noise suppression performance of this method outperforms the traditional algorithms. In the case of white Gaussian noise, we utilize MIMO sonar to detect multiple targets. If the elements of the transmit array and receive array are equaled spaced, then we need more elements to make up for the degree of freedom (DOF) loss caused by the virtual array that is overlapped. In order to improve the performance of the MUSIC algorithm, the theory and applications of time reversal (TR) are discussed [29]. TR-MUSIC was first applied to Born-approximated linear scattering model [30]. Further, the performance analysis and statistical testing of TR-MUSIC were provided by Ciuonzo et al. [31,32,33]. In particular, the hypothesis testing technique was adopted to propose a theoretically-founded decision statistics approach [32]. A low complexity MUSIC-based Toeplitz reconstruction method was utilized [34]. This method can effectively reduce the operation dimension and avoid the loss of virtual array aperture and DOF caused by the traditional decorrelation method. The dimension reduction transformation method and the ESPRIT algorithm are combined to reduce the calculation amount [35]. This method effectively reduces the complexity of the calculation and gains a better direction-finding result than the original algorithms. Further, this method was improved [36,37]. The addressing covariance reconstruction is used to do direction-finding of targets with the situation of an unknown target number [36]. The method proposed by Tan et al. [37] employs the beam space algorithm, which reduces the computational complexity and maintains the accuracy of the direction finding. However, the above three methods only consider the case of white Gaussian noise. They are not suitable for a complex marine noise environment. If we add colored noise to the echo signals, the accuracy of the DOA estimation cannot be guaranteed.

Therefore, an effective method to improve the noise suppression ability of multi-target DOA estimation is the main contribution of this paper. We proposed a new method for the accurate DOA estimation in the case of complex noise. The essence of this method is to improve the accuracy of direction finding, minimize the computational complexity and reduce the number of snapshots required. The ability to successfully reduce the number of snapshots is because this method is less sensitive to snapshots. It is worth mentioning that the probability of the target resolution of this method increases faster than traditional methods. Simultaneously, this method avoids the freedom loss caused by decoherent processing. We can simulate complex marine environments by adding colored noise to white Gaussian noise. In the actual noise field, the proportion of the symmetric noise component is much larger than that of the asymmetric noise component [38,39]. Considering that the imaginary part of the covariance matrix has no relationship with the symmetric noise component, we can remove the real part of the covariance matrix to avoid the effect of the symmetric noise component on DOA estimation. The real part of the covariance matrix is reconstructed by using the method of dimension reduction transformation and the replacement principle of the matrix imaginary component, and the interference of the bilateral spectrum interference is avoided. Thus, we obtain the reconstructed covariance matrix. Moreover, Toeplitz is utilized to reduce the correlation of the covariance matrix. This operation can reduce the influence of limited snapshots and complex marine noise on the covariance matrix. The new covariance matrix we obtained is closer to the ideal covariance matrix. Therefore, the phenomenon of fuzzy division between the signal subspace and the noise subspace can be avoided. To further reduce the computational complexity, dimension reduction processing is once again used to obtain a lower dimension covariance matrix. Above all, we get a novel noise suppression method for MIMO sonar DOA estimation. This method is based on dimension reduction transformation and the Toeplitz decoherence technique. For the sake of simplicity, we abbreviate this method as RC-STIM. Then, DOA estimation methods, such as MUSIC can be used to pre-estimate the target angle and carry out the final DOA estimation. Due to the established rotational invariance property at the MIMO sonar array, ESPRIT can be used to replace MUSIC. This search-free method can obviously reduce the computational complexity. In view of the advantageous property of MUSIC, we try to combine the advantages of the two methods to reach a balance between performance and complexity. As a result, the proposed algorithms can achieve better DOA estimation performance at lower computational cost, in limited snapshots, simultaneously.

The paper is organized as follows. In Section 2, the signal model of MIMO sonar is briefly introduced. Section 3 presents the influence of the noise component and the noise suppression MIMO sonar model. Several performance parameters, such as computational complexity, Cramer-Rao Bound and SNR gain are presented in Section 4. The simulation results which show the superiority of the proposed MUSIC-based and ESPRIT-based RC-STIM MIMO sonar DOA estimation techniques are presented in Section 5, followed by conclusions drawn in Section 6.

Notation: ${\left(\cdot \right)}^{T}$, ${\left(\cdot \right)}^{H}$, ${\left(\cdot \right)}^{C}$ and ${\left(\cdot \right)}^{-1}$ represent the transpose, conjugate-transpose, conjugate and inverse, respectively. ⊗ represents the Kronecker product. vec(⋅) indicates the vectorization operation. $I_{M\times M}$ denotes an M×M identity matrix, ${\mathbb{C}}^{M\times N}$ is M×N matrix. diag(⋅) represents the diagonalization operation.

## 2. MIMO Sonar Signal Model

**Figure 1:**
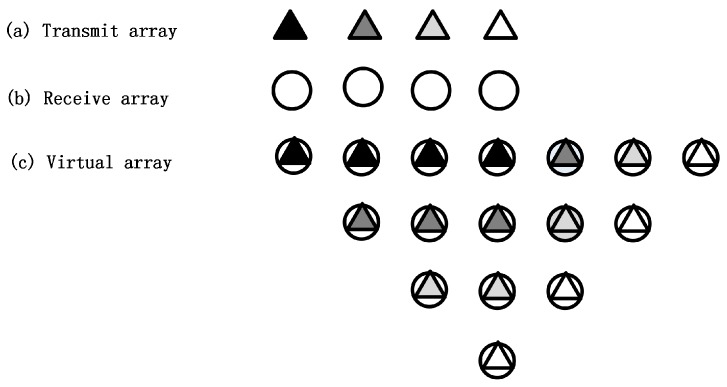
(**a**) The transmit array. (**b**) The receive array. (**c**) The MIMO sonar virtual array.

Consider a uniform linear array (ULA) of co-located MIMO sonar, equipped with a transmit array of M sensors and a receive array of N sensors, with half-wavelength spacing employed for both the transmit and receive arrays. The two arrays are collinear and the genetic centers are overlapped. The sensors are assumed to be in close proximity so that all the elements can detect a far-field target at the same spatial angle. Assume that there are K targets, satisfying the condition 2K<MN. $A_{T}=[a_{t}(\theta_{1}),a_{t}(\theta_{2}),\ldots ,a_{t}(\theta_{K})]$ is a M×K dimensional matrix composed of the K transmit array steering vector. $A_{R}(\theta )=[a_{r}(\theta_{1}),a_{r}(\theta_{2}),\ldots ,a_{r}(\theta_{K})]$ is a N×K dimensional matrix composed of the K receive array steering vector. The effectiveness of the proposed method is not influenced by whether the transmit and receive array element numbers are equal or not. For simplicity, but without loss of generality, take the co-located MIMO array with equal numbers of transmit and receive array elements as an example. The convolution operation is performed on the positions of the transmit and receive array elements [40], we can obtain the positions of the MIMO sonar virtual array elements, which are shown in Figure 1. Under the condition of far-field, the coordinates of each virtual array element are equal to the convolution of the corresponding transmit and receive array elements. The coordinates of the virtual array elements can be expressed as $VP_{i}=(m+n-1)d_{t},\;m=1,\ldots ,M,\;n=1,\ldots ,N$. In this scenario, several overlapped positions of virtual elements will appear due to the co-located location of the transmit and receive array elements. Each column in Figure 1 represents a set of virtual elements in the same location.

**Figure 2:**
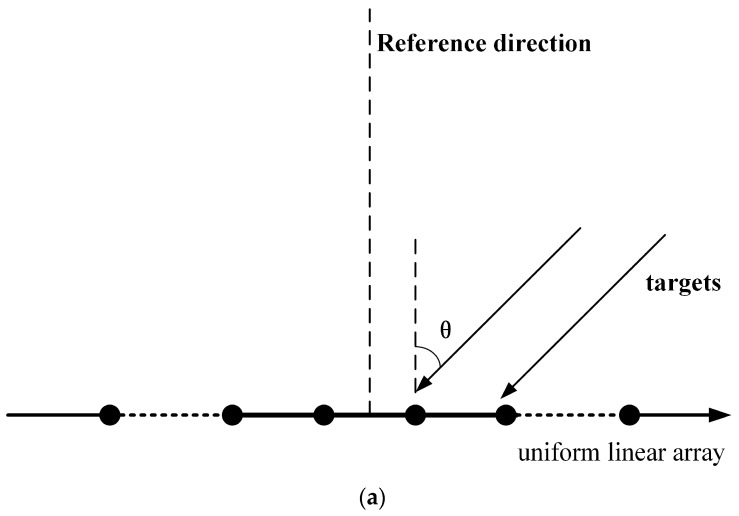

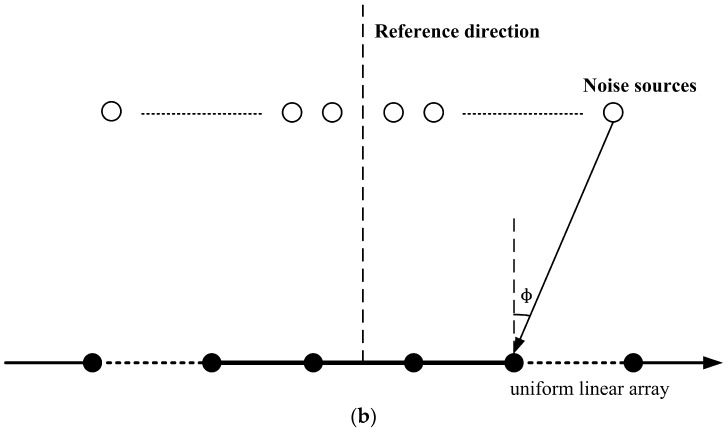
(**a**) The distribution model of the array elements and target signals. (**b**) The model of spatial noise.

The M transmit elements are used to transmit M orthogonal waveforms. Therefore, by discretization of P snapshots, the representation of the transmitted signals is a unit energy baseband pulse matrix. We assume that there is no Doppler shift between the targets and each element. Simultaneously, the effects of channel fluctuation, dielectric absorption, echo distortion and propagation loss on the echo signals are ignored. Then, we operate under the assumption that there is no multipath among the different sources’ emissions, i.e., the propagation is nondispersive. The narrowband echo signals of K far-field targets obtained at the receiver can be modeled as

(1) $$X=\sum_{k=1}^{K}\beta_{k}a_{r}(\theta_{k})a_{t}^{T}(\theta_{k})S+N$$

where $\beta_{k}$ is the reflection coefficient of the target located at an unknown spatial angle $\theta_{k}$. Spatial noise N, consisting of white Gaussian noise and colored noise, is a N×P dimensional matrix. ${\left(\;\;\right)}^{T}$ stands for transpose. The distribution model of array elements and target signals is plotted in Figure 2a. As can be seen from this figure, the vertical direction of the line array is the reference direction $0^{{}^{\circ}}$. The angle increases with rotating to the right until $90^{{}^{\circ}}$.

The components of spatial noise include white Gaussian noise and colored noise. It can be regarded as the superposition of discrete planar waves generated by several noise sources. Compared to actual noise, the error decreases as the number of noise sources increases. According to statistical characteristics, if we add as many noise sources as possible, we can gain an environmental noise field that is closer to the true value. As shown in Figure 2b, spatial noise can be decomposed into several narrowband sources. ϕ denotes the azimuth of the noise source. Assume that the spatial noise consists of Q narrowband noise sources with a frequency of f. The power and azimuth of the qth noise source is $\sigma_{q}^{2}$ and $\phi_{q}$, respectively. Obviously, we can get $\phi_{q}=\phi_{Q-q+1}$. The noise waveform is represented by $n_{q}(t)$. Therefore, we can obtain the noise waveform received by the mth array element: $\sum_{q=1}^{Q}n_{q}(t)e^{-jk_{n}^{T}(\phi_{q})P_{m}}$. Where $k_{n}(\phi_{q})=2\pi fv_{n}(\phi_{q})/c$, $v_{n}(\phi_{q})$ denotes the direction vector of the noise sources, c is the sound velocity, $P_{m}$ is the position vector of each element.

**Figure 3:**
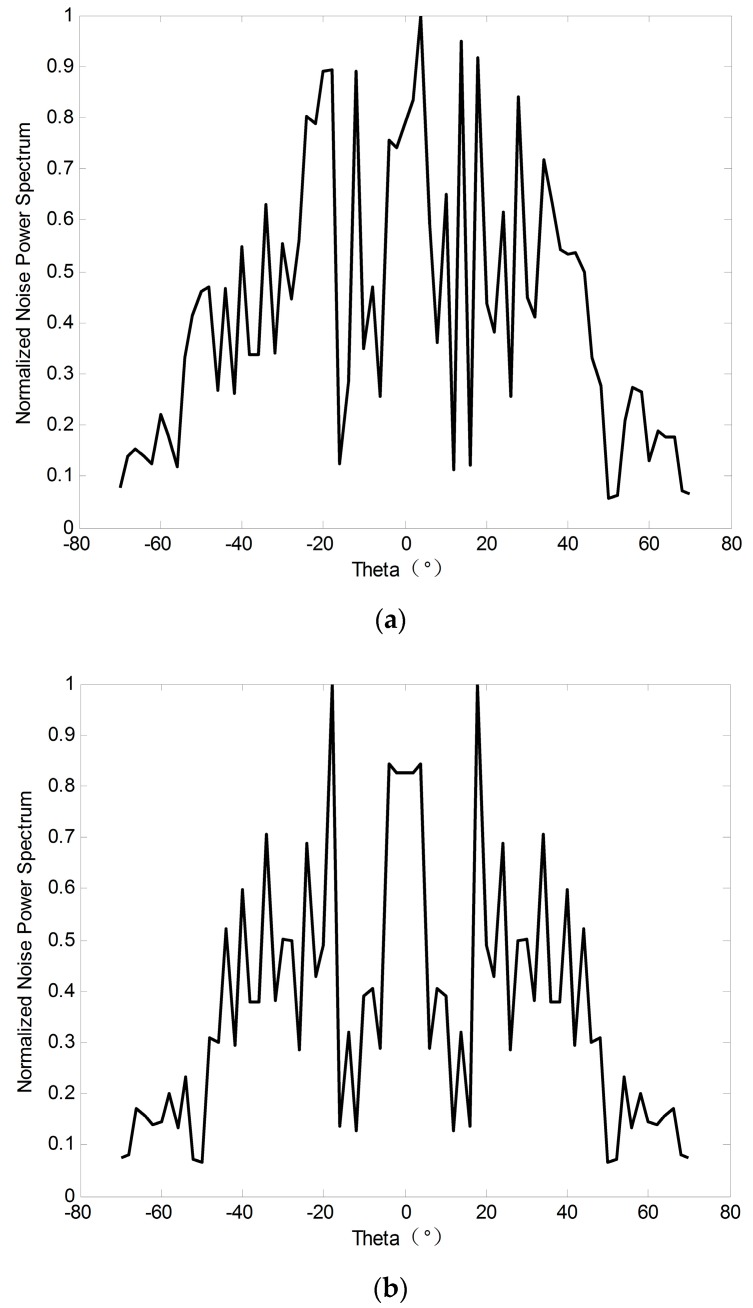
(**a**) The Noise Power Spectrum. (**b**) The Symmetric Noise Component.

For simplicity, we take two symmetric noise sources, $N_{1}$ and $N_{2}$ as examples. The azimuths are ϕ and −ϕ. The power is $\sigma_{N_{1}}^{2}$ and $\sigma_{N_{2}}^{2}$, $\sigma_{N_{1}}^{2}>\sigma_{N_{2}}^{2}$. We can get $\sigma_{N_{1}}^{2}=\sigma_{N_{2}}^{2}+\Delta_{\sigma_{N_{1}}^{2}}$, i.e., $N_{1}$ can be divided into $N_{1}^{\prime }$ with the power of $\sigma_{N_{2}}^{2}$ and $N_{\Delta }$ with the power of $\Delta_{\sigma_{N_{1}}^{2}}$. Therefore, we can define $N_{1}^{\prime }$ and $N_{2}$ as symmetric noise and define $N_{\Delta }$ as asymmetric noise. Further, we can draw the conclusion that random noise can be decomposed into a symmetric noise component and an asymmetric noise component. Figure 3 shows the power distribution and decomposition of the noise component. Therefore, subfigure (a) depicts the power of the noise component, it contains colored noise and white Gaussian noise term. Subfigure (b) shows the symmetric noise component. It can be seen from subfigure (b) that the azimuth and energy of noise are statistically symmetric around the receive array center. Obviously, we can draw that symmetrical noise component account for a much larger proportion than the asymmetric noise component; this may lead to a direct way of suppressing noise.

By using matched filtering on echo signals generated by the orthogonal signals S, we can obtain the autocorrelation function at the output end.

(2) $$\begin{array}{ll} \tilde{Y}=XS^{H} & =(\sum_{k=1}^{K}\beta_{k}a_{r}(\theta_{k})a_{t}^{T}(\theta_{k})S+N)S^{H}\\ & =\sum_{k=1}^{K}\beta_{k}a_{r}(\theta_{k})a_{t}^{T}(\theta_{k})(SS^{H})+NS^{H}, \end{array}$$

Because of the orthogonality between the echo signals, the covariance matrix of the transmitting signal can be simplified into the identity matrix $I_{M\times M}$. By substituting the matrix into Equation (2), the receive signal matrix can be reformulated as

(3) $${\tilde{Y}}^{H}=\sum_{k=1}^{K}\beta_{k}a_{r}(\theta_{k})a_{t}^{T}(\theta_{k})+NS^{H},$$

By vectorizing Equation (3), we can obtain the output end signal sample vector after matched filtering.

(4) $$\begin{array}{ll} y=\mathrm{vec}(\tilde{Y}) & =\mathrm{vec}(\sum_{k=1}^{K}\beta_{k}a_{r}(\theta_{k})a_{t}^{T}(\theta_{k})+NS^{H})\\ & =\sum_{k=1}^{K}\beta_{k}a_{tr}(\theta_{k})+n \end{array},$$

$a_{tr}(\theta_{k})=a_{t}(\theta_{k})⊗a_{r}(\theta_{k})=a_{r}(\theta_{k})a_{t}^{T}(\theta_{k})$, $n=n_{1}+n_{2}$, $n_{1}=\mathrm{vec}(N_{1}S^{H})$ obeys the spatial colored noise distribution. $n_{2}=\mathrm{vec}(N_{2}S^{H})$ obeys the complex Gaussian distribution with zero mean and covariance matrix $\sigma_{n}^{2}I_{MN}$. Set $A=[a_{tr}(\theta_{1}),a_{tr}(\theta_{2}),\ldots ,a_{tr}(\theta_{K})]$, $\beta ={\left[\beta_{1},\beta_{2},\ldots ,\beta_{K}\right]}^{T}$, Equation (4) can be expressed as

(5) y=Aβ+n,

The receiving signal matrix is composed of L snapshots.

(6) Y=[y(1),y(2),…,y(L)],

where B=[β(1),β(2),…,β(L)], W=[n(1),n(2),…,n(L)], Y can be expressed as a matrix form

(7) Y=AB+W,

By calculating the echo signal covariance matrix of Equation (7), we can obtain

(8) $$\begin{array}{ll} R_{YY} & =\frac{1}{L}\sum_{l=1}^{L}Y(l)Y{\left(l\right)}^{H}=AR_{BB}A^{H}+Q\\ & =R_{s}+R_{n} \end{array},$$

where Q is the covariance matrix of the spatial noise component, which contains spatial colored noise and white Gaussian noise. The uncorrelation between the target reflection coefficients enables simplifying the matrix into a diagonal matrix.

(9) $$R_{BB}=\frac{1}{L}\sum_{l=1}^{L}B(l)B^{H}(l)=\left[\begin{array}{cccc} \beta_{1}^{2} & & & \\ & \beta_{2}^{2} & & \\ & & \ddots & \\ & & & \beta_{K}^{2} \end{array}\right],$$

## 3. Problem Formulation

The main goal is to suppress the influence of noise in the direction of arrival estimation, especially the symmetric component of the spatial noise. In an actual acoustic environment, the symmetric noise component in the noise component has a large influence on the real part of the covariance matrix and has little effect on the imaginary part. By using the imaginary part DOA estimation method, the real part of the echo signal covariance matrix is removed, and the influence of symmetric noise on the direction-finding performance of the algorithm is reduced. To further analyze the covariance matrix, we consider the idea of dimension reduction transformation and obtain

(10) $$a_{tr}(\theta_{k})=Gd(\theta_{k}),$$

(11) $$d(\theta_{k})={\left[1,e^{-j\pi \sin(\theta_{k})},\ldots ,e^{-j\pi (M+N-2)\sin(\theta_{k})}\right]}^{T},$$

(12) $$G=\left[\begin{array}{l} \begin{array}{lllllll} 1 & 0 & \cdots & 0 & 0 & \cdots & 0\\ 0 & 1 & \cdots & 0 & 0 & \cdots & 0\\ \vdots & \vdots & \ddots & \vdots & \vdots & \ddots & \vdots \\ 0 & 0 & \cdots & 1 & 0 & \cdots & 0 \end{array}\}M\\ \begin{array}{lllllll} 0 & 1 & 0 & \cdots & 0 & \cdots & 0\\ 0 & 0 & 1 & \cdots & 0 & \cdots & 0\\ \vdots & \vdots & \vdots & \ddots & \vdots & \ddots & \vdots \\ 0 & 0 & 0 & \cdots & 1 & \cdots & 0 \end{array}\}M\\ \begin{array}{lllllll} 0 & \cdots & 0 & 1 & 0 & \cdots & 0\\ 0 & \cdots & 0 & 0 & 1 & \cdots & 0\\ \vdots & \ddots & \vdots & \vdots & \vdots & \ddots & \vdots \\ 0 & 0 & 0 & 0 & 0 & \cdots & 1 \end{array}\}M \end{array}\right]\in {\mathbb{C}}^{MN\times (M+N-1)},$$

It can be rewritten as a matrix

(13) $$\begin{array}{l} A=GD\\ D=[d(\theta_{k}),d(\theta_{k}),\cdots ,d(\theta_{k})]\in {\mathbb{C}}^{(M+N-1)\times K} \end{array},$$

The signal covariance matrix $R_{s}$ can be modeled as

(14) $$\begin{array}{l} R_{s}=AR_{BB}A^{H}=G(D\left[\begin{array}{cccc} \beta_{1}^{2} & & & \\ & \beta_{2}^{2} & & \\ & & \ddots & \\ & & & \beta_{K}^{2} \end{array}\right]D^{H})G^{H}\\ \;\;\;\;=G\left[\begin{array}{cccc} \sum_{k=1}^{K}\beta_{k}^{2} & \sum_{k=1}^{K}\beta_{k}^{2}e^{j\pi \sin\theta_{k}} & \cdots & \sum_{k=1}^{K}\beta_{k}^{2}e^{j\pi (M+N-2)\sin\theta_{k}}\\ \sum_{k=1}^{K}\beta_{k}^{2}e^{j\pi \sin\theta_{k}} & \sum_{k=1}^{K}\beta_{k}^{2} & \cdots & \sum_{k=1}^{K}\beta_{k}^{2}e^{j\pi (M+N-3)\sin\theta_{k}}\\ \vdots & \vdots & \ddots & \vdots \\ \sum_{k=1}^{K}\beta_{k}^{2}e^{j\pi (M+N-2)\sin\theta_{k}} & \sum_{k=1}^{K}\beta_{k}^{2}e^{j\pi (M+N-3)\sin\theta_{k}} & \cdots & \sum_{k=1}^{K}\beta_{k}^{2} \end{array}\right]G^{H} \end{array},$$

The signal covariance matrix can be expressed as ${\left(R_{s}\right)}_{a,b}=G(\sum_{k=1}^{K}\beta_{k}^{2}e^{j\pi (b-a)\sin\theta_{k}})G^{H}$; the imaginary part of the signal covariance matrix can be obtained as

(15) $$\begin{array}{ll} {\left(R_{si}\right)}_{a,b} & =jG\left[\sum_{k=1}^{K}\beta_{k}^{2}\sin(j\pi (b-a)\sin\theta_{k})\right]G^{H}\\ & =\frac{1}{2}G\left[\sum_{k=1}^{K}\beta_{k}^{2}e^{j\pi (b-a)\sin\theta_{k}}\right]G^{H}-\frac{1}{2}G\left[\sum_{k=1}^{K}\beta_{k}^{2}e^{j\pi (b-a)\sin(-\theta_{k})}\right]G^{H} \end{array},$$

Considering Equation (15), the two parts of the equation can be regarded as the covariance matrix of the signal matrix with the incident angle of $\theta_{k}$ and $-\theta_{k}$. It is worth noting that the imaginary part of the signal covariance matrix contains the correct target direction-finding information and the false target direction-finding information. The two sets of azimuths are symmetric about the normal direction of the array.

The noise component can be considered as the superposition of mutually independent plane waves generated by several noise sources. The larger the number of noise sources, the closer the noise is to the real noise. In this paper, we decompose the spatial noise component into several narrowband noises with the quantity of P and the center frequency of f. We can rewrite the noise as

(16) $$n=\sum_{p=1}^{P}a_{n}(\varphi_{p})n_{p}(t),$$

To simplify the calculation, in Figure 2, we assume that there are only two receive array elements and two symmetric noise sources, each of which is independent with each other and has the same power $\delta^{2}$. The azimuth angle of the two noise sources relative to the array normal is θ and −θ. The output signals with matched filtering of the two receive array elements can be respectively modeled as

(17) $$X_{1}(t)=N_{1}(t)S^{H}(t)+N_{2}(t)S^{H}(t),$$

(18) $$X_{2}(t)=N_{1}(t)e^{-j\pi \sin\theta }S^{H}(t)+N_{2}(t)e^{j\pi \sin\theta }S^{H}(t),$$

The spatial correlation function of $X_{1}(t)$ and $X_{2}(t)$ can be expressed as

(19) $$\gamma_{12}=E\left[X_{1}(t)X_{2}^{H}(t)\right]=\delta_{s}^{2}E({\left|N_{1}(t)\right|}^{2})e^{j\pi \sin\theta }+\delta_{s}^{2}E({\left|N_{2}(t)\right|}^{2})e^{-j\pi \sin\theta },$$

where $E({\left|S(t)\right|}^{2})=\delta_{s}^{2}$, $E({\left|N_{1}(t)\right|}^{2})=E({\left|N_{2}(t)\right|}^{2})=\delta^{2}$. Equation (19) can be simplified as

(20) $$\gamma_{12}=\delta_{s}^{2}\delta^{2}e^{j\pi \sin\theta }+\delta_{s}^{2}\delta^{2}e^{-j\pi \sin\theta }=2\delta_{s}^{2}\delta^{2}\cos(\pi \sin\theta ),$$

It is worth noting that the imaginary part of $\gamma_{12}$ is zero. Therefore, the covariance matrix of the symmetric noise component is a real symmetric matrix. Moreover, the imaginary part of the covariance matrix is independent of the symmetric noise components in the noise component. From Equation (20), we observe that the interference of symmetric noise components in the noise component can be suppressed by eliminating the real part of the covariance matrix.

Let the real part of the covariance matrix $R_{YY}$ is $R_{R}$, and the imaginary part is $R_{I}$. Suppose that $\Lambda =[A,A^{c}]$. We can define a new matrix

(21) $$\begin{array}{ll} \Delta R & =R_{YY}-R_{YY}^{c}\\ & =\Lambda \left[\begin{array}{cc} R_{BB} & 0\\ 0 & -R_{BB} \end{array}\right]\Lambda^{H} \end{array},$$

where ${\left(\;\;\right)}^{c}$ denotes conjugate. As $\left[A,A^{c}\right]$ and $\mathrm{diag}(R_{BB},-R_{BB})$ are all full column rank matrices and the rank is 2K. Therefore, the rank of ΔR is 2K. The power spectrum obtained from the imaginary part contains 2K peaks, where K pseudo-peaks are symmetric with the real peaks. The angles of the peaks are expressed as $\Psi =[\psi_{1},\psi_{2},\ldots ,\psi_{K},-\psi_{1},-\psi_{2},\ldots ,-\psi_{K}]$, the real angle estimation is $\Theta =[\theta_{1},\theta_{2},\ldots ,\theta_{K},-\theta_{1},-\theta_{2},\ldots ,-\theta_{K}]$. Among them, the first K elements are the real target locations. Suppose that the matrix I is the permutation matrix generated by noise, the locations of each element in Θ are changed by one transformation and we can get the following relationship Ψ=ΘI. By constructing the array manifold matrix of the imaginary part by $A_{T}^{c}(\theta )=[a_{r}^{c}(\theta_{1}),a_{r}^{c}(\theta_{2}),\ldots ,a_{r}^{c}(\theta_{K})]$, $A_{I}(\psi )=[a_{r}(\psi_{1}),a_{r}(\psi_{2}),\ldots ,a_{r}(\psi_{K}),a_{r}^{c}(\psi_{1}),a_{r}^{c}(\psi_{2}),\ldots ,a_{r}^{c}(\psi_{K})]$ is obtained, so $A_{I}=\Lambda I$. Thus, we can build matrix

(22) $$\tilde{R}={\left(A_{I}^{H}A_{I}\right)}^{-1}A_{I}^{H}\Delta RA_{I}{\left(A_{I}^{H}A_{I}\right)}^{-1},$$

By substituting Equation (21) into Equation (22), we can get

(23) $$\begin{array}{ll} \tilde{R} & ={\left(A_{I}^{H}A_{I}\right)}^{-1}A_{I}^{H}\Lambda \left[\begin{array}{cc} R_{BB} & 0\\ 0 & -R_{BB} \end{array}\right]\Lambda^{H}A_{I}{\left(A_{I}^{H}A_{I}\right)}^{-1}\\ & =I^{T}\left[\begin{array}{cc} R_{BB} & 0\\ 0 & -R_{BB} \end{array}\right]I \end{array},$$

when $A_{I}=\Lambda$, we can get I=E. The diagonal elements in $\tilde{R}$ are sorted correctly. In this scenario, the first K diagonal elements are positive and the corresponding K target reflected signal power. When $A_{I}\neq \Lambda$, the diagonal sort is not correct in $\tilde{R}$. The matrix is still a diagonal matrix after two transformations, but the positions of the elements on the diagonal change. The positive signal power estimated value corresponds to the correct signal angle. By confirming the correct signal angle estimation and signal power estimation according to the element positions of the diagonal in Equation (23), we extract the positive value of the diagonal in $\tilde{R}$ as the signal power ${\tilde{\sigma }}_{k}^{2}\;(k=1,2,\cdots ,K)$. The angle information in Ψ corresponding to the position is the target direction of the estimation. Construct the estimated power matrix containing the signal as $\Delta_{s}=\mathrm{diag}\left({\tilde{\sigma }}_{1}^{2},{\tilde{\sigma }}_{2}^{2},\ldots ,{\tilde{\sigma }}_{K}^{2}\right)$. By considering the idea of dimension reduction transformation, we can obtain $a_{tr}(\psi_{k})=Gd(\psi_{k})$, $d(\psi_{k})={\left[1,e^{-j\pi \sin(\psi_{k})},\ldots ,e^{-j\pi (M+N-2)\sin(\psi_{k})}\right]}^{T}$, which can be rewritten as a matrix $A=GD,\;D=[d(\psi_{1}),d(\psi_{2}),\cdots ,d(\psi_{K})]\in {\mathbb{C}}^{(M+N-1)\times K}$. We reconstruct the covariance matrix as

(24) $$\hat{R}=A\Delta_{s}A^{H}=(GD)\Delta_{s}{\left(GD\right)}^{H}=G(D\Delta_{s}D^{H})G^{H},$$

By taking the real part of $D\Delta_{s}D^{H}$, we can obtain $R_{\Delta }={\left(r_{ij}\right)}_{i,j=1,2,\ldots M+N-1}$, $r_{ij}=\sum_{k=1}^{K}\sigma_{k}^{2}\cos[(j-i)\pi \sin\psi_{k}]$. Therefore, the covariance matrix of the echo signal with noise suppression can be reconstructed as

(25) $$R=GR_{\Delta }G^{H}+jR_{I},$$

Because of the numerical difference and complex noise in the array elements, the covariance matrix is an approximate Toeplitz matrix. In this case, when we do eigen-decomposition on the covariance matrix, the division of the signal subspace and noise subspace is not clear, which will lead to a performance decline in the DOA estimation of MUSIC algorithm. This phenomenon is particularly pronounced in the case of low SNR, the direction-finding result shows worse performance compared with the traditional MUSIC method. Therefore, we can modify the real part $R_{R}$ and imaginary part $R_{I}$ of the reconstructed covariance matrix R by Toeplitz respectively.

By averaging the diagonal elements of the matrix $R_{\Delta }$, we can obtain the covariance matrix of the ideal situation. Symbol m and n denote the rows and columns of M+N−1 dimension matrix, $r_{mn}$ denotes the element of the mth row and the nth column. The modified matrix elements can be expressed as

(26) $$\begin{array}{l} {\hat{r}}_{mn}=\hat{r}(m-n)=\frac{1}{M+N-1-(m-n)}\sum_{i=1}^{M+N-1-(m-n)}{\hat{r}}_{i(i+m-n)}\;\;\;m\leq n\\ {\hat{r}}_{mn}=\hat{r}(m-n)=\hat{r}(-(m-n))={\hat{r}}_{mn}^{H}\;\;\;m>n \end{array}$$

We can obtain the modified $R_{R}$ by Equation (25). In the same way, by averaging the diagonal elements of matrix $R_{I}$, we can obtain the modified $R_{I}$. However, it is worth noting that, since the MIMO sonar system normally has a large virtual array number, the computational complexity of the conventional subspace-based method grows up greatly. We use the reduced dimension transformation method to improve computational efficiency. Based on Equation (12), we define $W=G^{H}G$, and get $W=diag(1,2,\cdots ,\underset{\left|M-N\right|+1}{\underset{︸}{\min(M,N),\cdots ,\min(M,N)}},\cdots ,2,1)$. We denote $P=W^{-\frac{1}{2}}G^{H}$ by the reduced dimension transformation method and then use the transformation P for the echo signal after matched filtering. We can obtain the dimension reduction matrix z(t)=Py(t). Moreover, the covariance matrix $R_{z}$ can be expressed as

(27) $$\begin{array}{ll} R_{z} & =E[z(t)z^{H}(t)]\\ & =PE[y(t)y^{H}(t)]P^{H} \end{array}$$

Through the above transformation, we obtain the covariance matrix $R_{z}$, which efficiently reduces the computational complexity. It is worth pointing that since the application of the spatial spectrum estimation algorithm is in the DOA estimation, it is necessary to know the number of signal sources. The signal sources can be estimated according to the distribution of the covariance matrix eigenvalues [41]. On the other hand, considering the MIMO sonar configuration and the dimension reduction transformation, the DOF of the proposed method is DOF=M+N−2, i.e., the number of detectable signal sources.

Up to now, we have achieved the noise suppression method for DOA estimation in co-located MIMO sonar. We show the major steps of the proposed method as follows:

- Remove the real part of the covariance matrix $R_{YY}$. The imaginary part $R_{I}$ is used for DOA pre-estimation.
- Construct G matrix, then use the sorting refactoring method and the dimension reduction transformation to reconstruct the real part of $R_{YY}$. We can obtain the new covariance matrix R.
- Reduce the coherence of R by the Toeplitz method.
- Construct W and P from G, and then use P left multiply R and use $P^{H}$ right multiply R. Through the dimension reduction transformation, we can get the covariance matrix $R_{z}$.
- Compute the noise subspace and the signal subspace, finally estimating DOAs.

The choice of the twice DOA estimation methods in the steps will be discussed in the following section.

## 4. Performance Analysis

In this section, we analyze the performance of the noise suppression technique for MIMO sonar DOA estimation. For simplicity, RC-STIM is used to represent the noise suppression method we proposed.

In the following subsection, we assume a uniform linear transmit array consists of M=8 omnidirectional sensors. The spacing of the elements is half a wavelength. The receive array is the same as the transmit array; the centers of the two arrays are overlapped. We compare the aforementioned methods in terms of the computational complexity, Cramer-Rao bound (CRB) and SNR gain methods.

### 4.1. Computational Complexity Analysis

The RC-STIM method we proposed needs to be combined with the appropriate DOA estimation algorithm to complete direction-finding. Considering the characteristics of the MIMO sonar array, we adopt the MUSIC algorithm and the ESPRIT algorithm. For achieving the effect of suppressing noise, we need to perform two DOA estimation operations. In order to obtain the computational complexity of the proposed algorithm, we can analyze the complexity of the traditional MUSIC algorithm (hereinafter referred to as MUSIC). The computational complexity of the MUSIC algorithm is mainly affected by the covariance matrix calculation, eigenvalue decomposition (EVD) and spectral peak search calculation. Compared with the MUSIC algorithm, the proposed method requires higher computational complexity. On the other hand, the dimension reduction transformation can help to reduce the computational complexity. For convenience, we abbreviate the MUSIC-based dimension reduction method as RC-MUSIC. While $O\{{\left(M+N-1\right)}^{2}L+{\left(M+N-1\right)}^{3}+n[(M+N-1)(M+N-1-K)+M+N-1-K]\}$ presents the computational complexity of RC-MUSIC. $O\left\{M^{2}N^{2}L+M^{3}N^{3}+n[MN(MN-K)+MN-K]\right\}$ presents the MUSIC method which needs higher computational cost than RC-MUSIC. n is the number of searching steps. Hence, the total computational complexity of the proposed method is

(28) $$\begin{array}{l} O\{M^{2}N^{2}L+M^{3}N^{3}+n[MN(MN-K)+MN-K]+{\left(M+N-1\right)}^{2}K\\ +{\left(M+N-1\right)}^{3}+n[(M+N-1)(M+N-1-K)+M+N-1-K]\} \end{array}$$

Similarly, we can get the computational complexity of the ESPRIT-based RC-STIM algorithm. The abbreviated definition of RC-ESPRIT is the same as above. Different from the MUSIC algorithm, the ESPRIT algorithm avoids the computational load brought by spectral peak search and the calculating speed is more prominent than the MUSIC-based methods. Hence, the total computational complexity of the proposed method is

(29) $$O\{M^{2}N^{2}L+M^{3}N^{3}+2K^{3}+{\left(M+N-1\right)}^{2}K+{\left(M+N-1\right)}^{3}\}$$

while RC-ESPRIT requires $O\{{\left(M+N-1\right)}^{2}L+{\left(M+N-1\right)}^{3}+K^{3}\}$. ESPRIT requires $O\{M^{2}N^{2}L+M^{3}N^{3}+K^{3}\}$. Through all these equations, we can see that the ESPRIT-based methods outperform the MUSIC-based methods in computational complexity. In order to achieve noise suppression, we perform DOA pre-estimation, which leads to an increase in computational complexity. In this case, we use the dimension reduction transformation and search-free direction-finding techniques to further reduce the computational complexity.

### 4.2. Cramer-Rao Bound

In our framework, a useful statistical bound for evaluating the limiting DOA estimation performance is the CRB. In this section, we analyze the CRB of MIMO sonar DOA estimation accuracy. It can be derived from the considered reconstructed covariance matrix, i.e., the model expressed by Equation (27). It is worth mentioning that the proposed MIMO sonar model with the reconstructed covariance matrix differs from the traditional MIMO sonar model given in Equation (4) due to the fact that the reduced dimension transformation method is used in Equation (27). Because of the irrelevance of noise and signals, the covariance matrix of noise can be written as

(30) $$Q=E\left[n(t)n^{H}(t)\right]=\mathrm{diag}(\sigma_{1}^{2},\sigma_{2}^{2},\cdots ,\sigma_{MN}^{2})$$

According to Refs. [42,43], we derive the CRB estimation as

(31) CRB=12L{Re[(D˜H∏A˜⊥D˜)⊙PT]}−1

where $\tilde{A}=Q^{-\frac{1}{2}}A$, $\prod_{\tilde{A}}^{⊥}=I_{MN}-\tilde{A}{\left({\tilde{A}}^{H}\tilde{A}\right)}^{-1}{\tilde{A}}^{H}$; $\tilde{D}=Q^{-\frac{1}{2}}D$, $D=\left[d_{1},d_{2},\cdots ,d_{K}\right]$, $d_{k}=\partial a_{tr}(\theta_{k})/\partial \theta_{k}$; $P=\frac{1}{L}\sum_{l=1}^{L}s(t_{l})s^{H}(t_{l})$ and ⊙ stands for the Schur–Hadamard matrix product.

### 4.3. SNR Gain

Assume that the noise source signals that generate colored noise are irrelevant, therefore, the covariance matrix of colored noise can be modeled as γ and we assume that the imaginary part of the main diagonal is $\mathrm{diag}(\gamma_{1},\gamma_{2},\ldots ,\gamma_{MN})$. We can obtain the SNR of the received signals from Equation (8)

(32) $${\mathrm{SNR}}_{i}=\frac{\mathrm{tr}(R_{s})}{\mathrm{tr}(I_{MN}\sigma^{2}+\gamma )}=\frac{\mathrm{tr}(R_{s})}{MN\sigma^{2}+\sum_{i=1}^{MN}\gamma_{i}}$$

The SNR after dimension reduction can be obtained from Equation (27). It is worth noting that the processing of the covariance matrix reconstruction helps to reduce the symmetric noise component. Hence, we can obtain

(33) $${\mathrm{SNR}}_{o}=\frac{\mathrm{tr}(W^{-\frac{1}{2}}G^{H}R_{s}GW^{-\frac{1}{2}})}{\mathrm{tr}(\gamma )}=\frac{\mathrm{tr}(R_{s}GW^{-\frac{1}{2}}G^{H})}{\sum_{i=1}^{M+N-1}\gamma_{i}}$$

We can achieve the extra SNR gain from the dimension reduction processing.

(34) $$g=\frac{SNR_{o}}{SNR_{i}}=\frac{\mathrm{tr}(R_{s}GW^{-\frac{1}{2}}G^{H})\left[MN\sigma^{2}+\sum_{i=1}^{MN}\gamma_{i}\right]}{\left(\sum_{i=1}^{M+N-1}\gamma_{i}\right)\mathrm{tr}(R_{s})}=\frac{MN\sigma^{2}+\sum_{i=1}^{MN}\gamma_{i}}{\sum_{i=1}^{M+N-1}\gamma_{i}}$$

## 5. Simulation Results

Throughout our simulations, we adopt the co-located MIMO array. We assume that the transmit and receive arrays are both uniform linear arrays with half wavelength spacing of each element and M=N=8. Suppose that the direction of the two targets are $\theta_{1}=-15^{{}^{\circ}},\theta_{2}=20^{{}^{\circ}}$. The additive noise is spatial colored noise and the production process has been shown in Figure 3. We use several simulations to compare the performances of the proposed DOA methods, especially MUSIC and ESPRIT.

In this paper, we use the proposed covariance matrix reconstruction and dimension reduction method to improve the noise suppression ability. At this time, the subspace decomposition-based techniques such as MUSIC and the search-free direction-finding techniques such as ESPRIT can be used for multiple targets’ DOA estimation. In all simulations, unless otherwise stated, all methods are computed based on 500 independent runs.

We evaluate the DOA estimation performance of our algorithms and present the root-mean-square error (RMSE) as

(35) $$\mathrm{RMSE}=\frac{1}{\mathrm{MONT}}\sum_{n=1}^{\mathrm{MONT}}\sqrt{\frac{1}{K}\sum_{k=1}^{K}{\left({\hat{\theta }}_{k}^{\left(n\right)}-\theta_{k}\right)}^{2}}$$

where MONT denotes the number of Monte Carlo experiments, ${\hat{\theta }}_{k}^{\left(n\right)}$ denotes the estimated angle in the nth experiment.

**Figure 4:**
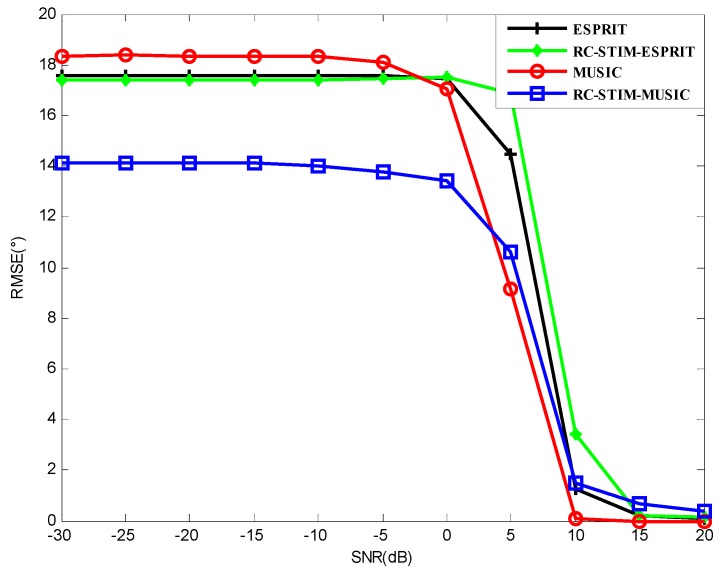
The root mean square error (RMSE) versus the signal-to-noise ratio (SNR) for the MUSIC-based and ESPRIT-based direction-of-arrival (DOA) estimation in a spatial colored noise environment.

Figure 4 shows the RMSE for the MUSIC-based and ESPRIT-based DOA estimators versus SNR for all the method test results. The noise environment is spatial colored noise and white Gaussian noise. A total number of snapshots L=50 is used. It can be seen from this figure that the MUSIC-based MIMO sonar with the proposed RC-STIM method outperforms the traditional MIMO sonar MUSIC algorithm at the low SNR region while the opposite occurs at the high SNR region, the same situation to the other two methods based on ESPRIT. This means that the traditional algorithm is more sensitive to colored noise at the low SNR region, while the covariance matrix reconstruction operation helps to reduce the influence of the symmetric colored noise part on the DOA estimation. It can also be observed that the two methods based on ESPRIT show a similar DOA estimation accuracy. This is because under limited snapshots, the estimation performance is limited by ESPRIT. In practice, if we need to achieve a balance between the computational complexity and the DOA estimation accuracy, we can combine the MUSIC and ESPRIT algorithms, this will be considered in the following section.

**Figure 5:**
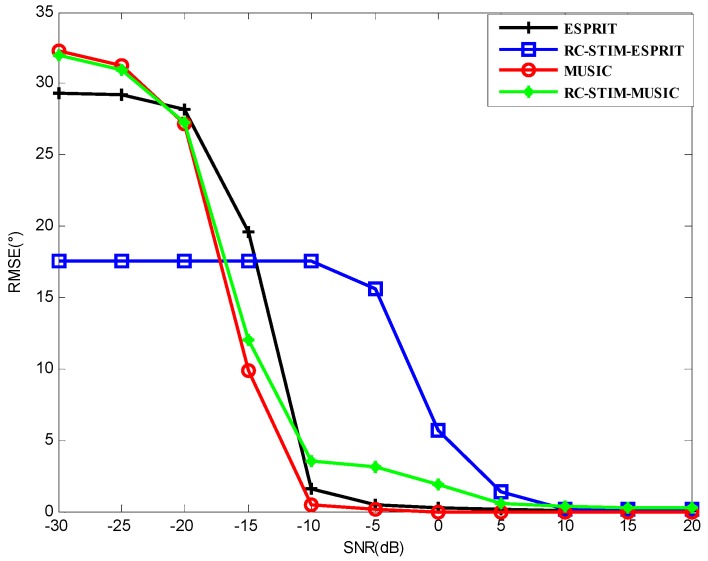
The RMSE versus the SNR for MUSIC-based and ESPRIT-based DOA estimation in a spatial white Gaussian noise environment.

It can be seen from Figure 5 that in the white Gaussian noise environment the noise suppression performance of ESPRIT-based RC-STIM algorithm is prominent at the low SNR region, while the superiority of MUSIC-based algorithms is feasible at the high SNR region. Therefore, the ESPRIT-based algorithms can be selected when the white Gaussian noise is the main noise component. It can also be observed from Figure 5 that the covariance matrix reconstruction and dimension reduction processing can efficiently reduce the sensitivity of the algorithm to white Gaussian noise.

**Figure 6:**
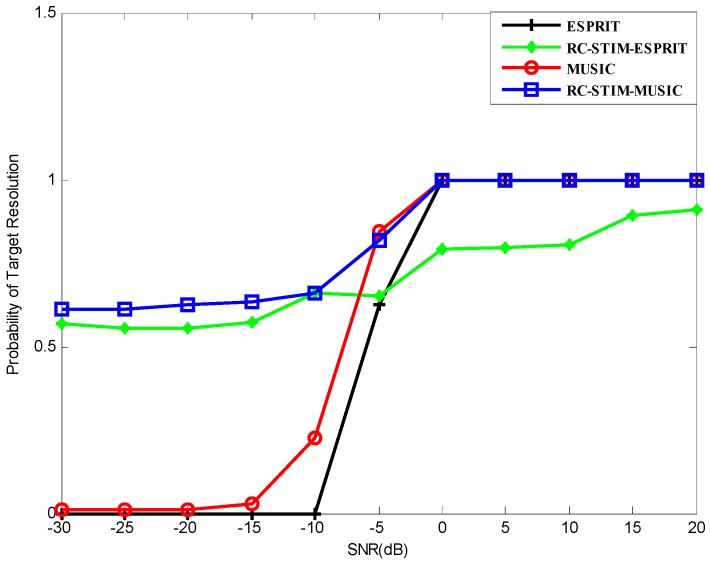
The probability of the target resolution versus the SNR for MUSIC-based and ESPRIT-based DOA estimators.

Furthermore, we consider that the DOA estimation is considered to be solved when the following is satisfied

(36) $$\left|{\hat{\theta }}_{k}-\theta_{k}\right|\leq \frac{\Delta \theta }{2},\;\;\;k=1,2,\ldots ,K$$

where $\Delta \theta =\left|\theta_{2}-\theta_{1}\right|$, and ${\hat{\theta }}_{k}$ denotes the estimation of $\theta_{k}$. The source resolution probability versus SNR for all the methods we proposed is shown in Figure 6. It can be seen from the figure that the probability of the source resolution for each method starts to grow at a certain point as the SNR increases. All methods, except the ESPRIT-based RC-STIM method, exhibit a 100% correct source resolution probability at a high SNR value. It can be seen from Figure 6 that the ESPRIT-based RC-STIM method has the highest SNR threshold. This is because the direction-finding precision of the ESPRIT-based algorithms is obviously affected by the preliminary DOA estimation processing and the limitation of algorithm DOA estimation accuracy will influence the reconstruction of the covariance matrix. The SNR thresholds of the ESPRIT method, the MUSIC method and the MUSIC-based RC-STIM method are lower than the aforementioned method, at about 0 dB. Moreover, except for the two RC-STIM algorithms, the target resolution probability of the remaining two algorithms is close to zero as SNR decreases to −10 dB and −20 dB. The ESPRIT-based RC-STIM algorithm is better than these two methods, the target resolution probability will not decrease to zero. Finally, the MUSIC-based RC-STIM method remains at a target resolution probability above 50%, i.e., the best performance of the source resolution probability.

It is worth noticing that, in the case of low SNR, the MUSIC-based RC-STIM algorithm has a strong suppression of colored noise, while the ESPRIT-based RC-STIM algorithm can effectively reduce the impact of the white Gaussian noise. Considering the computational complexity and noise suppression, we can try to combine the proposed two methods. The computational complexity of the proposed methods has been discussed above. To access a higher DOA estimation accuracy and lower computational complexity to improve the algorithm performance, we utilize ESPRIT to carry out the preliminary DOA estimation before covariance matrix construction processing, and take the advantage of MUSIC in final DOA estimation to ensure the target direction-finding precision. Another option is to reverse the two methods. From the above, the computational complexity of this method is

(37) $$\begin{array}{l} O\{M^{2}N^{2}L+M^{3}N^{3}+K^{3}+{\left(M+N-1\right)}^{2}K\\ +{\left(M+N-1\right)}^{3}+n[(M+N-1)(M+N-1-K)+M+N-1-K]\} \end{array}$$

(38) $$O\left\{M^{2}N^{2}L+M^{3}N^{3}+n[MN(MN-K)+MN-K]+{\left(M+N-1\right)}^{2}K+{\left(M+N-1\right)}^{3}+K^{3}\right\}$$

**Figure 7:**
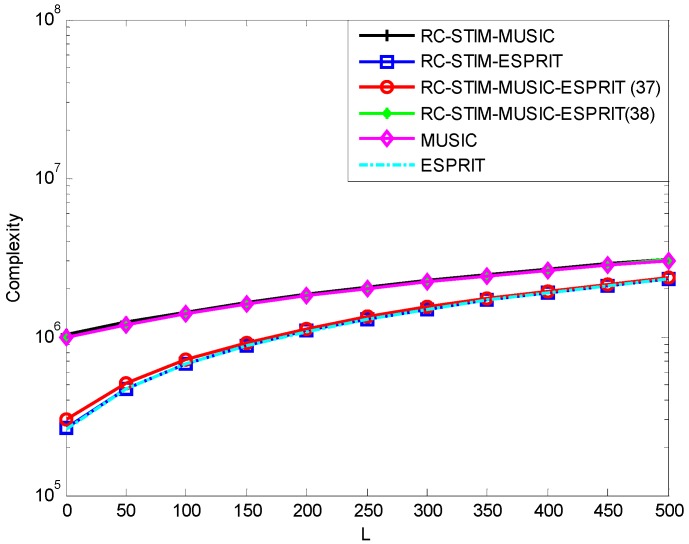
The computational complexity versus snapshot number for MUSIC-based and ESPRIT-based DOA estimators.

Figure 7 shows the computational complexity of the MUSIC-based and ESPRIT-based DOA estimations. It can be seen from this figure that the MUSIC-ESPRIT-based method of Equation (37) with a covariance matrix construction operation and dimension reduction processing has an advantage in computational complexity, the computational load is similar to the ESPRIT-based methods. Moreover, with the increasing of snapshots, this advantage will gradually decrease. Therefore, under limited snapshot conditions, the performance loss due to a limited snapshot number is proportional to the covariance matrix dimension. The lower the covariance matrix dimension, the less performance loss. As a result, the method we proposed gains better performance than traditional algorithms versus the limited snapshot number. On the other hand, the method of Equation (38) shows a slight advantage in improving computational complexity. However, this method better preserves the performance of MUSIC algorithm in the preliminary DOA estimation. In the processing of preliminary DOA estimation, the precision of direction-finding directly affects the accuracy of covariance matrix reconstruction.

**Figure 8:**
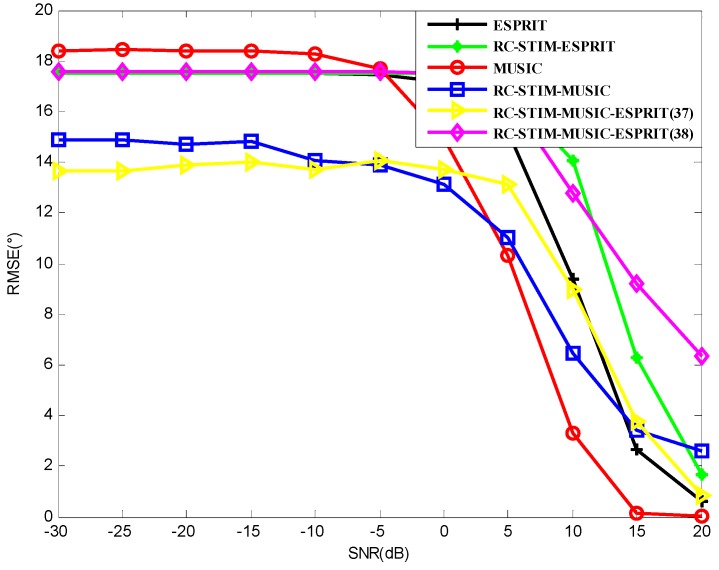
The RMSE versus SNR for MUSIC-based and ESPRIT-based DOA estimation in a spatial colored noise environment.

**Figure 9:**
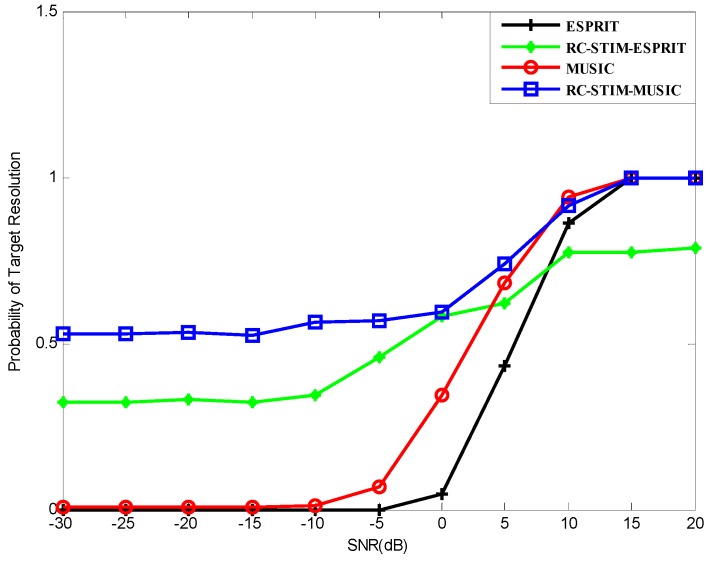
The probability of the target resolution versus SNR for MUSIC-based and ESPRIT-based DOA estimators.

Since the computational complexity of the algorithms is related to the snapshots, we reduce the snapshots to L=10. In this way, we can observe the effect of the snapshots on the proposed algorithms. Figure 8 shows the RMSE versus SNR respectively. Compared with Figure 4, the RMSE of the four corresponding algorithms in Figure 8 is not significantly different. Therefore, the snapshots have little effect on the DOA estimation accuracy of the algorithms. Obviously, Method (37) shows better performance than Method (38). In the low signal area, Method (37) outperforms all the other methods. With the increase of SNR, Method (37) gradually lost this advantage. The target resolution probability of the four algorithms in Figure 6 in the condition of L=10 are shown in Figure 9. In this figure, the target resolution probabilities of the shown methods are all reduced, compared with Figure 6. The ESPRIT-based RC-STIM method failed to reach a 100% correct source resolution at SNR=20 dB. Meanwhile, the SNR threshold of the remaining three algorithms is about 15 dB, which is much higher than the situation in Figure 6. The target resolution probability of the two traditional algorithms decreases to 0 at SNR=−10 dB and SNR=−5 dB. The ESPRIT-based RC-STIM method shows better performance, which will not go down to 0. The MUSIC-based RC-STIM method outperforms all the other methods. In brief, the target resolution probability of all the methods are affected by snapshots, meanwhile, the RC-STIM methods show a lower sensitivity to snapshots.

**Figure 10:**
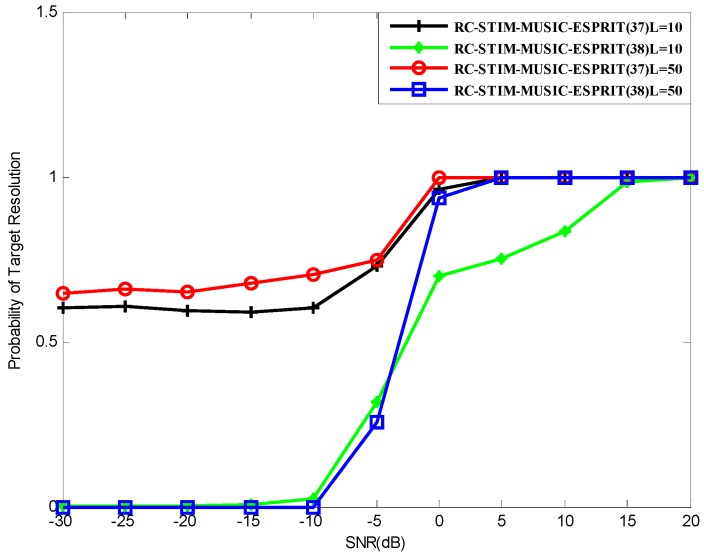
The probability of the target resolution versus SNR for Methods (37) and (38) in the case of different snapshot number.

Figure 10 shows the target resolution probability of Methods (37) and (38). For comparison, we consider different snapshots, i.e., L=10 and L=50. Method (37) has better performance compared with Method (38), regardless of the value of snapshot. Method (37) successfully reaches a 100% correct source resolution at SNR=5 dB, both at L=10 and L=50. This situation is obviously better than the algorithms in Figure 9, however, it is slightly worse than the algorithms in Figure 6. This means that the performance of Method (37) is not sensitive to changes of snapshots, which helps to provide better performance in limited snapshot conditions. Meanwhile, the target resolution probability of Method (38) is not sensitive to snapshots in a low SNR situation. As SNR increases, the SNR threshold of L=50 is obviously better than L=10. Compared to the aforementioned four algorithms, the SNR threshold of Method (38) is higher at snapshots L=50, and basically consistent with them in limited snapshots. As a result, Method (37) outperforms all the mentioned methods in limited snapshots.

**Figure 11:**
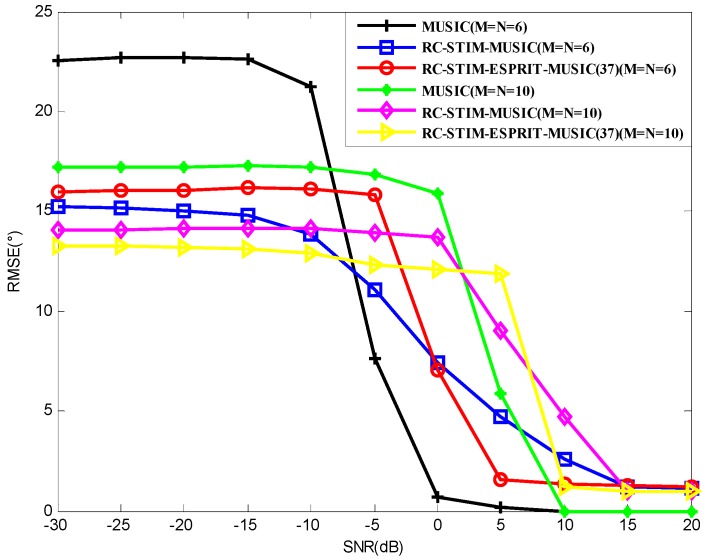
The RMSE versus SNR for MUSIC-based and ESPRIT-based DOA estimation in the case of different elements.

Figure 11 presents the effect of array element number on the performance of the algorithms. Obviously, the performance of the algorithm is proportional to the number of array elements, i.e., for the same algorithm, the larger the number of array elements, the higher the direction-finding accuracy of this algorithm. This situation can be attributed to the diversity gain. In the scenario of M=N=6 and low SNR, the MUSIC-based RC-STIM method outperforms the other two methods. As the SNR increases, when SNR reaches about −5 dB, we can gain better DOA estimation through the traditional MUSIC method. However, under the condition of M=N=10, Method (37) has the best DOA estimation accuracy, until the SNR is greater than −5 dB.

## 6. Conclusions

The influence of symmetric noise component on algorithm performance for multiple targets DOA estimation is proposed. A group of methods for noise suppression is introduced. The essence of the aforementioned methods is covariance matrix reconstruction and dimension reduction. Unlike the previous methods that directly utilize the covariance matrix to do DOA estimation, a priori processing, i.e., pre-estimation, is used to decrease the effect of the symmetric noise component. Dimension reduction processing can help to reduce the computational complexity. MUSIC and ESPRIT can then be used for multiple targets’ DOA estimation. The ESPRIT-based methods are more advantageous in terms of reducing computational complexity. Simultaneously, Method (37) allows us to achieve a better DOA estimation performance in the lower SNR region. Performance analysis of the proposed noise suppression methods and comparison to the existing MIMO sonar techniques are given. The computational complexity of the proposed methods can be controlled by the selection of the snapshots and the prior DOA estimation algorithm. The performance of the MUSIC-based RC-STIM method and Method (37) outperform the other methods. Simulation results show the superiority of the two proposed methods over the existing methods.

In future work, we intend to further optimize the noise suppression algorithm model, reduce the impact of the pre-estimation result on the DOA estimation accuracy. On the other hand, the pre-estimation result can be utilized to design the transmit beam space to improve SNR. Thus, the DOA estimation accuracy can be improved.
